# Whole-Genome Sequences of Eight *Campylobacter jejuni* Isolates from Wild Birds

**DOI:** 10.1128/genomeA.00315-15

**Published:** 2015-04-23

**Authors:** Anselme Shyaka, Akiko Kusumoto, Hiroshi Asakura, Keiko Kawamoto

**Affiliations:** a Section of Food Microbiology and Immunology, Diagnostic Center for Animal Health and Food Safety, Obihiro University of Agriculture and Veterinary Medicine, Obihiro, Japan; b Department of Veterinary Medicine, College of Agriculture and Veterinary Medicine, University of Rwanda, the College of Agriculture and Veterinary Medicine, Butare, Rwanda; c Division of Biomedical Food Research, National Institute of Health Sciences, Tokyo, Japan

## Abstract

We present here the draft genome sequences of 8 *Campylobacter jejuni* strains isolated from wild birds. The strains were initially isolated from swabs taken from resident wild birds in the Tokachi area of Japan. The genome sizes range from 1.65 to 1.77 Mbp.

## GENOME ANNOUNCEMENT

*Campylobacter jejuni* is a prominent cause of bacterial food poisoning worldwide (1). It is mainly transmitted to humans through the consumption of contaminated poultry, meat, milk, or water, although other pathways, including through wild birds, have been suggested (2–6). *C. jejuni* can colonize multiple hosts, including wild birds (7–14), and its genome can acquire exogenous genetic materials, leading to the genomic diversity of this bacterium (15–17). To understand its genetic acquisition and virulence mechanisms in various hosts, it is important to have the genomes from these different hosts. However, there are no genomes available yet from wild birds.

In this study, a total of 8 *C. jejuni* isolates, obtained from wild birds, were sequenced. Four isolates (designated *C. jejuni* C1-5523, C2-5524, C10-448, and C12-5524) were isolated from crows (*Corvus corona* and *Corvus macrorhynchos*), and four others (*C. jejuni* P3-2209, P5-2209, P9-2209, and P10-2209) were from pigeons (*Streptopelia orientalis* and *Columba livia*). The isolates were analyzed using multilocus sequence typing (MLST). The results showed that two are novel sequence types (ST), namely, ST5523, assigned to C1-5523, and ST5524, found in two isolates, C2-5524 and C12-5524. C10-448 was assigned to ST448, which was previously reported in various sources, including humans (http://www.pubmlst.org). Moreover, isolates from pigeons belong to the ST2209 of the clonal complex ST179 found in wild birds and humans (18).

Genomic DNA was extracted from a bacterial culture using the DNeasy blood and tissue kit (Qiagen, Hilden, Germany), and a genomic DNA (gDNA) sequencing library was prepared using the Nextera XT sample preparation kit (Illumina, San Diego, CA), according to the manufacturer’s instructions. The isolates were sequenced at our laboratory using a MiSeq (Illumina), with a read length of 150 bp.

Paired sequences comprising 1,435,488, 3,540,662, 3,839,026, 2,968,922, 2,338,804, 2,275,220, 1,659,742, and 2,773,330 bp were obtained from C1-5523, C2-5524, C10-448, C12-5524, P3-2209, P5-2209, P9-2209, and P10-2209, respectively. The reads were assembled using CLC Genomics Workbench (CLC bio, Aarhus, Denmark), and contigs of <200 bp were discarded. The characteristics of the 8 draft genome assembly and annotations from the RAST server (19) are shown in Table 1.

**TABLE 1:** Genome characteristics and accession numbers for eight *C. jejuni* isolates from wild birds

Isolate no.	No. of contigs	Fold coverage	G+C content (%)	Estimated genome length (bp)	*N*_50_ after scaffolding (bp)	Largest contig size (bp)	No. of genes by RAST	GenBank accession no.	Version accession no.
C1-5523	132	79	30.7	1,737,665	37,086	146,205	1,779	JYDX00000000	JYDX01000000
C2-5524	79	242	30.3	1,741,143	87,990	183,591	1,827	JXTR00000000	JXTR01000000
C10-448	79	231	30.1	1,775,512	107,221	186,605	1,879	JYDY00000000	JYDY01000000
C12-5524	79	207	30.3	1,748,582	71,617	191,218	1,846	JYDZ00000000	JYDZ01000000
P3-2209	77	147	30.9	1,679,025	153,531	331,256	1,719	JYEA00000000	JYEA01000000
P5-2209	50	191	30.7	1,664,340	147,170	225,387	1,718	JYEB00000000	JYEB01000000
P9-2209	70	133	30.7	1,659,064	69,932	198,306	1,708	JXTS00000000	JXTS01000000
P10-2209	74	183	30.7	1,669,250	96,955	200,111	1,719	JYEC00000000	JYEC01000000

Based on Mauve (20) alignments (data not shown), genomes from wild birds share large syntenic regions with that of *C. jejuni* strain NCTC 11168. However, we have found that C1-5523, C2-5524, and C12-5524 have acquired a *tetO* gene coding for resistance to tetracycline. Moreover, prophage-like elements similar to CJIE1 and CJIE4 of RM1221 (21) were identified in C2-5524, C10-448, and C12-5524. A type VI secretion system in C10-448 was also identified; however, further studies should be done to confirm its functionality. Interestingly, isolates from pigeons harbor a Cj0742 homologue, a filamentous hemagglutination domain protein that is longer than the normally truncated one found in NCTC 11168, implying a possible high host cell binding ability for these isolates (22).

In conclusion, these draft genomes will provide further insights into the genomic diversity of *C. jejuni* in determining the unique features of these isolates from wild birds, as well as a better understanding of its potential threat to humans.

### Nucleotide sequence accession numbers. 

These whole-genome sequences have been deposited in GenBank under the accession numbers listed in Table 1.
